# Correlation of Lung Damage on CT Scan with Laboratory Inflammatory Markers in COVID-19 Patients: A Single-Center Study from Romania

**DOI:** 10.3390/jcm11154299

**Published:** 2022-07-25

**Authors:** Cosmin Citu, Oana Maria Gorun, Andrei Motoc, Ioana Mihaela Citu, Florin Gorun, Daniel Malita

**Affiliations:** 1 Department of Obstetrics and Gynecology, “Victor Babes” University of Medicine and Pharmacy Timisoara, 2 Eftimie Murgu Square, 300041 Timisoara, Romania; citu.ioan@umft.ro (C.C.); gorun.florin@umft.ro (F.G.); 2 Department of Obstetrics and Gynecology, Municipal Emergency Clinical Hospital Timisoara, 1-3 Alexandru Odobescu Street, 300202 Timisoara, Romania; 3 Department of Anatomy and Embryology, “Victor Babes” University of Medicine and Pharmacy Timisoara, 2 Eftimie Murgu Square, 300041 Timisoara, Romania; amotoc@umft.ro; 4 Department of Internal Medicine I, “Victor Babes” University of Medicine and Pharmacy Timisoara, 2 Eftimie Murgu Square, 300041 Timisoara, Romania; citu.ioana@umft.ro; 5 Department of Radiology, “Victor Babes” University of Medicine and Pharmacy, 2 Eftimie Murgu Square, 300041 Timisoara, Romania; malita.daniel@umft.ro

**Keywords:** COVID-19, inflammatory markers, computed tomography

## Abstract

(1) Background: This study aims to evaluate the association of CRP, NLR, IL-6, and Procalcitonin with lung damage observed on CT scans; (2) Methods: A cross-sectional study was performed among 106 COVID-19 patients hospitalized in Timisoara Municipal Emergency Hospital. Chest CT and laboratory analysis were performed in all patients. The rank Spearmen correlation was used to assess the association between inflammatory markers and lung involvement. In addition, ROC curve analysis was used to determine the accuracy of inflammatory markers in the diagnosis of severe lung damage; (3) Results: CRP, NLR, and IL-6 were significantly positively correlated with lung damage. All inflammatory markers had good accuracy for diagnosis of severe lung involvement. Moreover, IL-6 has the highest AUC- ROC curve; (4) Conclusions: The inflammatory markers are associated with lung damage and can be used to evaluate COVID-19 severity.

## 1. Introduction

Coronavirus disease 2019 (COVID-19) is an infectious respiratory illness caused by severe acute respiratory syndrome coronavirus 2 (SARS-CoV-2) that spread rapidly, causing the COVID-19 pandemic. The outbreak of this disease was recorded in late December 2019 when several cases of atypical pneumonia caused by an unknown virus were reported in Wuhan, Hubei province, China [1]. Patients with COVID-19 may be asymptomatic or, in the most severe cases, may develop pneumonia that can lead to respiratory failure, respiratory distress syndrome, and ultimately death. It has been almost two years since COVID-19 was declared a pandemic by the World Health Organization [2]. In all this time, COVID-19 has caused hundreds of millions of infections and millions of deaths worldwide. In Romania, by 19 February 2022, more than 2 million infections and over 62,000 deaths were recorded [3]. COVID-19 is diagnosed by reverse transcription-polymerase chain reaction (RT-PCR) to detect SARS CoV-2 nucleic acid in a sample of nasopharyngeal (NS) swab.

However, due to inadequate clinical samples, differences in the detection rates of different RT-PCR kits and low patient viral load, the RT-PCR sensitivity for COVID-19 infection is about 71% [4]. Chest CT (computed tomography) is a non-invasive and rapid method that can provide essential information on the diagnosis and evolution of COVID-19 pneumonia [5,6,7,8]. Several studies have reported CT patterns and features including ground-glass opacity (GGO), consolidation, and pleural effusion [9]. In addition, an important method of assessing the severity of patients with COVID-19 is to measure markers of inflammation [10,11]. In this study, we aimed to evaluate in patients with COVID-19 in Romania the association between lung injury on CT scans and changes in inflammatory markers such as CRP (C-reactive protein), IL-6 (interleukin 6), procalcitonin, and NLR (neutrophil to lymphocyte ratio). The relationship between the severity of coronavirus infection and various markers of inflammation is well established in many papers. CT is used in the assessment of COVID-19 severity in combination with conventional tests. However, in many areas of our country and globally, CT assessment is impossible due to logistical problems and weak health systems. Conventional tests are much cheaper and easier to use. Thus, this paper proposes to demonstrate that the lung damage in COVID-19 may be determined by assessing inflammatory markers, with a perspective of using these tests to classify critically ill patients in hospitals that are not equipped with CT.

## 2. Materials and Methods

### 2.1. Study Design and Setting

A single-center, cross-sectional study was conducted among patients with COVID-19 diagnosed and treated at the Timisoara Municipal Emergency Hospital between 15 August 2021, and 15 October 2021.

Diagnosis of SARS-COV-2 infection was confirmed by RT-PCR according to the national guidelines issued by the National Institute of Public Health of Romania.

This study was approved by the ethics committee of the University of Medicine and Pharmacy “Victor Babes” Timisoara (no. 22726/17 November 2021).

### 2.2. Participants

Participants included met the following criteria: (1) Sars-CoV-2 infection confirmed by a real-time reverse transcriptase-polymerase chain reaction test; (2) optimal chest CT scan performed on admission; (3) no other known infections; (4) no known lung tumors; and (5) no pregnancy. Bacterial co-infection was excluded based on microbiological cultures. None of the included participants self-administered anti-inflammatory or antiviral drugs prior to admission.

### 2.3. Variables and Data Collection

The data for analysis were collected from the electronic medical record system of the Clinical Municipal Emergency Hospital of Timisoara. The following data were collected for each patient: (1) demographic information; (2) vital signs: temperature (°-Celsius), oxygen saturation in room air, respiratory rate (per minute); (3) laboratory data: white blood cell (WBC), lymphocyte count, platelet, neutrophils count, monocyte count, C-reactive protein (CRP), interleukin 6 (Il-6), and procalcitonin; (4) radiologic findings; and (5) clinical course: length of hospitalization and death. In addition, the neutrophil/lymphocyte ratio (NLR) was calculated as a simple ratio between the number of neutrophils and lymphocytes. Laboratory data, vital signs, and radiological findings included in the analysis were obtained on admission. The severity of COVID-19 was classified as non-severe and severe disease. Patients were defined as presenting severe disease if they had one of the following syndromes: (1) severe pneumonia defined by fever plus one of: respiratory rate > 30 breaths/min, severe respiratory distress, or SpO_2_ ≤ 93% on room air; or (2) acute respiratory distress syndrome (ARDS) which was defined by new or worsening respiratory symptoms with impaired oxygenation (PaO_2_/FiO_2_a ≤ 300 mmHg), bilateral opacities, not fully explained by volume overload, lobar or lung collapse, or nodules on chest imaging [12].

Therefore, patients were included in the severe disease group if they had one of the following: SpO_2_ < 94%, PaO_2_/FiO_2_ < 300 mm Hg, a respiratory rate > 30 breaths/min, or pulmonary infiltrates > 50% [12,13].

### 2.4. Chest CT Imaging

All initial chest CT scans were performed on the day of patient admission using a GE BrightSpeed 16 Slice CT Scanner (GE Healthcare). Visual assessment of CT scans was performed by several different experienced radiologists. Radiologists were blind to the clinical signs and symptoms presented by patients at the time of CT scan assessment.

Computed tomography scanning guidelines included the degree of total parenchymal lung involvement and the presence of CT features such as ground-glass opacification (GGO), consolidations, and pleural effusion.

CT images were classified, using a simple score, according to the percentage of the entire lung parenchyma affected by COVID-19 lesions into two groups: non-severe pulmonary damage (<50%) and severe pulmonary damage (≥50%).

### 2.5. Statistical Analysis

RStudio (R version 4.2.0: A language and environment for statistical computing. R Foundation for Statistical Computing, Vienna, Austria. https://www.R-project.org/) was used for data analysis. Categorical variables were presented in numbers (percentage) and were compared using Fisher’s exact test. Continuous variables were presented in medians [interquartile range] and were compared using the Mann–Whitney U test.

Correlations were assessed using the Spearman rank correlation coefficient. Grading standards were: no correlation (r = 0), very weak correlation (r = 0–0.19), weak correlation (r = 0.20–0.39), moderate correlation (r = 0.40–0.59), strong correlation (0.60–0.79), very strong correlation (0.80–1.00).

The area under the receiver operating characteristics curve was used to evaluate the diagnostic accuracy of the inflammatory biomarkers in the determination of lung damage above 50%. Youden index was used for the determination of optimal cut-offs. Binary logistic regression was used to determine the predictive value of inflammatory markers for severe lung injury on CT. Covariates used to adjust odds ratios were age, comorbidities, and gender; *p* < 0.05 was considered statistically significant.

## 3. Results

### 3.1. Characteristics of the Patients

In this study, a total of 106 patients hospitalized with confirmed COVID-19 were included (Table 1). The median age was 69 years, and the majority of patients were women (54.7%). The most common comorbidity present was hypertension (71.6%) followed by chronic kidney disease (62.3%). The in-hospital mortality rate was 26.4% with no statistically significant difference between the severe COVID-19 and non-severe COVID-19 groups (*p* = 0.07). Patients with severe disease had a longer period of hospitalization (median = 11) compared to those with non-severe disease (median = 9). However, this difference is not statistically significant (*p* = 0.59). Regarding the CT scan findings, ground-glass opacities were found in 64.2% of patients, and the median lung involvement was 25(IQR = 15–40). In addition, regarding the severity of lung damage assessed on CT, 14 patients had severe lung involvement (≥50%), and 92 patients had non-severe lung lesions (<50%).

### 3.2. Association of Inflammatory Biomarkers with Lung Injury Severity in COVID-19

**Figure 1:**
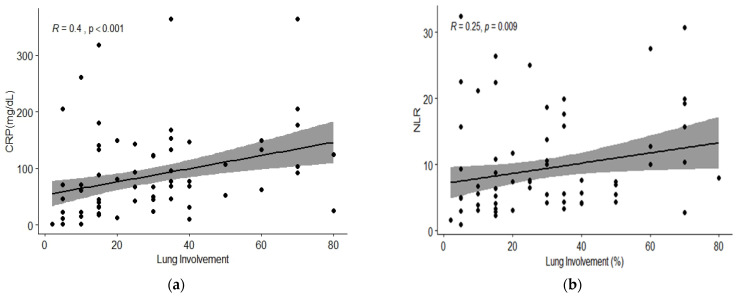
Correlation of chest CT lung involvement with different inflammatory biomarkers: (**a**) C-reactive protein (mg/dL); (**b**) neutrophil to lymphocyte ratio. On the x-axis, the lung damage in percent (continuous scale) is represented and on the y-axis the value of CRP and NLR respectively.

**Figure 2:**
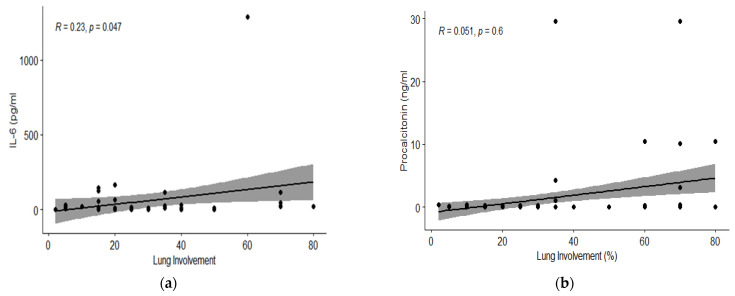
Correlation of chest CT lung involvement with different inflammatory biomarkers: (**a**) interleukin-6 (pg/mL); (**b**) Procalcitonin (ng/mL). On the x-axis the lung damage in percent (continuous scale) is represented and on the y-axis the value of IL-6 and procalcitonin respectively.

Spearman’s rank correlation was computed to assess the relationship between blood inflammatory markers and lung involvement on CT scans. There was a positive correlation between CRP and NLR, respectively, and IL-6 and lung damage on CT (r = 0.40, *p* < 0.001; r= 0.25, *p* = 0.009, respectively, r = 0.23, *p* = 0.04) (Figure 1 and Figure 2). Procalcitonin showed no statistically significant correlation with lung damage (r = 0.05, *p* = 0.66) (Figure 2).

**Figure 3:**
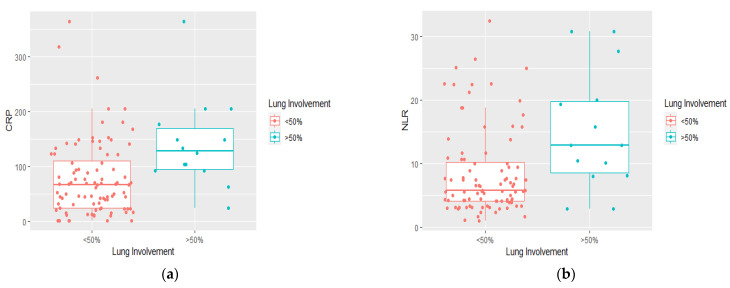
The difference in medians of inflammatory markers according to lung involvement on CT scan on admission: (**a**) C-reactive protein (mg/dL); (**b**) neutrophil to lymphocyte ratio. The x-axis represents the classification of lung involvement: <50% (non-severe), >50% (severe). On the y-axis the value of CRP and NLR, respectively.

**Figure 4:**
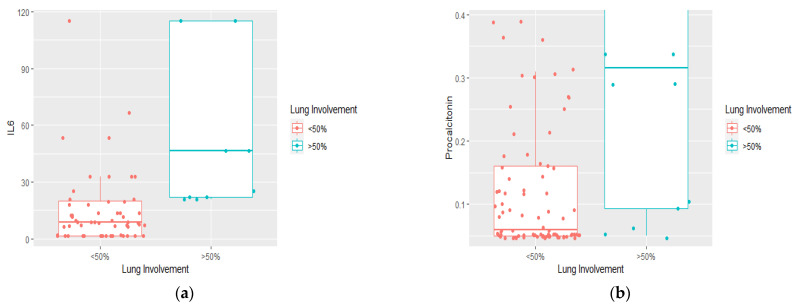
Difference in medians of inflammatory markers according to lung involvement on CT scan on admission: (**a**) interleukin-6 (pg/mL); (**b**) procalcitonin (ng/mL). The x-axis represents the classification of lung involvement: <50% (non-severe), >50% (severe). On the y-axis the value of IL-6 and procalcitonin, respectively.

The median values of CRP, NLR, IL-6, and procalcitonin were: 67.7, 5.70, 8.66, respectively, 0.06 for the group with lung involvement below 50%, and 129.2, 12.8, 46.49, respectively, 0.31, for the group with lung involvement above 50% (Figure 3 and Figure 4). CRP, NLR, IL-6, and procalcitonin values were statistically significantly higher in patients with lung involvement above 50% compared to patients with lung involvement below 50% (*p* = 0.001, *p* = 0.006, *p* < 0.001, and *p* = 0.001, respectively).

### 3.3. Receiver Operating Characteristic Analysis of Inflammatory Biomarkers on Admission

**Figure 5:**
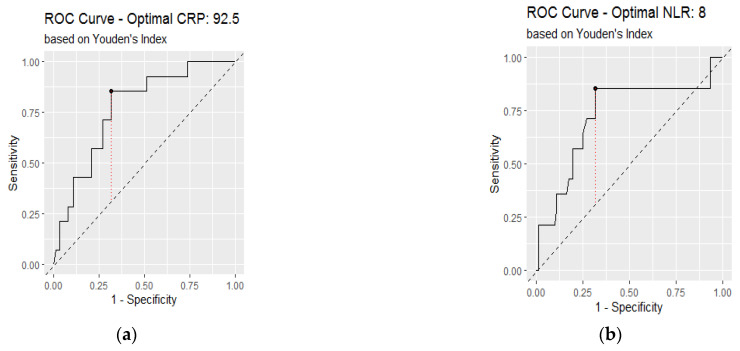
Receiver operating characteristic curve analysis to assess the diagnostic value of inflammatory biomarkers for lung involvement over 50%: (**a**) C-reactive protein; (**b**) neutrophil to lymphocyte ratio.

**Figure 6:**
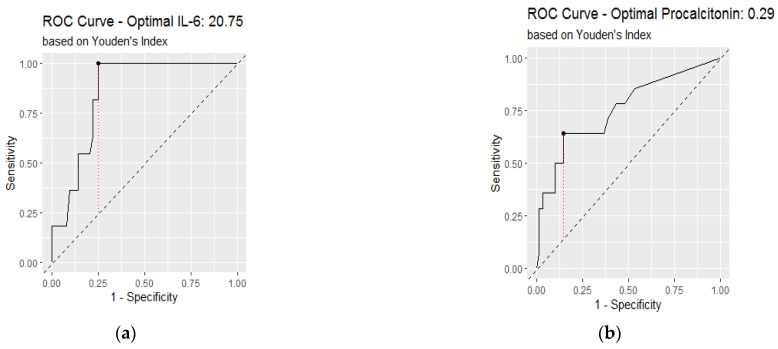
Receiver operating characteristic curve analysis to assess the diagnostic value of inflammatory biomarkers for lung involvement over 50%: (**a**) interleukin-6; (**b**) procalcitonin.

The area under the curve (AUC) was calculated to determine the accuracy of inflammatory markers in diagnosing lung involvement above 50% (Figure 5 and Figure 6). The areas under the curve of CRP, NLR, IL-6, and procalcitonin were above 0.7 (Table 2). The highest accuracy was found in IL-6 with an AUC of 0.854.

Table 2 shows the optimal cut-off for inflammatory markers obtained by the Youden index. The highest sensitivity was found for IL-6 (100%) and the highest specificity for procalcitonin (85%).

In addition, a binomial logistic regression was performed to test the discriminatory ability of inflammatory markers (above or below cutoff values) as prognostic factors for severe lung injury on CT, adjusted for age, comorbidities, and gender. Results showed an aOR of 9.32 for CRP above 92.5 mg/dL, of 5.73 for NLR above 8, of 43.2 for IL-6 above 20.7 pg/mL and 18.71 for procalcitonin above 0.29 ng/mL (Table 3).

**Figure 7:**
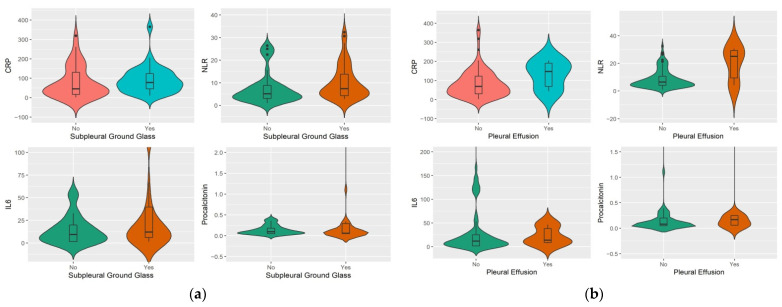
Difference in medians of inflammatory markers by presence on CT: (**a**) subpleural ground glass; (**b**) pleural effusion.

Furthermore, NLR values were statistically significantly higher in patients with subpleural ground-glass findings on CT (median = 7.40 vs. 5.12, *p* = 0.02). However, CRP, IL6 and procalcitonin values were not significantly higher in these patients (*p* = 0.06, *p* = 0.10, and *p* = 0.83) (Figure 7). Additionally, NLR values were also significantly increased in patients with pleural effusion (25.0 vs. 6.5, *p* = 0.04) (Figure 7). Although CRP, IL6, and procalcitonin values were higher in these patients, the differences were not statistically significant (*p* = 0.09, *p* = 0.52, and *p* = 0.53).

**Figure 8:**
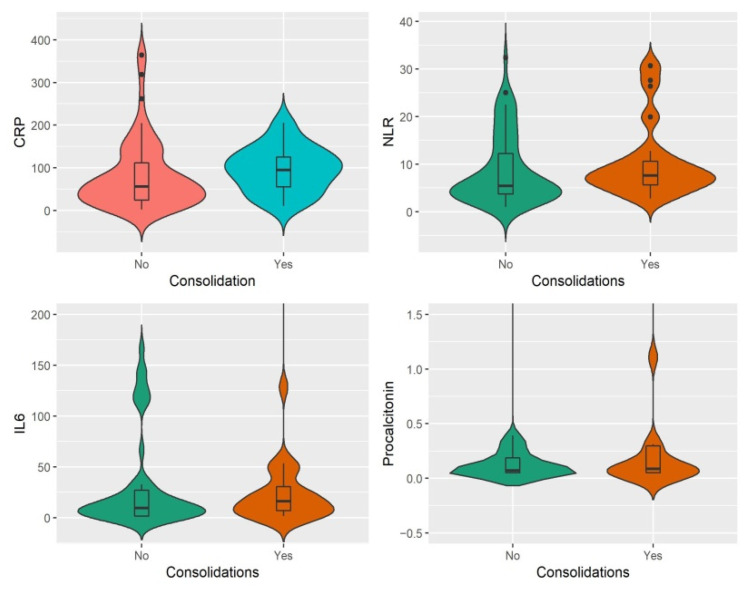
Difference between the medians of inflammatory markers according to CT presence of consolidations.

As for patients with findings of consolidation on CT, these had significantly higher CRP and NLR values (94.9 vs. 55.8, *p* = 0.03, respectively, 7.6 vs. 5.4, *p* = 0.04) (Figure 8).

## 4. Discussion

In our study, we collected laboratory data and CT findings from 106 COVID-19 patients admitted to the Timisoara Municipal Emergency Clinical Hospital between 15 August 2021, and 15 October 2021. The present research revealed that there are correlations between chest CT features of patients with COVID-19 and changes in CRP, procalcitonin, IL-6, and NLR parameters; thus the latter can be used to assess disease severity.

Of the changes following chest CT scans, the most common imaging manifestation was ground-glass opacity (64.2%). Various studies have similarly reported that GGO is the most common change on CT images, followed by consolidation [14,15,16,17,18,19]. Pleural effusion, along with pericardial effusion, lymphadenopathy, cavitation, and pneumothorax are among the less common but potential features found as the disease progresses [20]. In addition, from our results, a low incidence of 5.66 % of cases had pleural effusion on a chest CT scan, belonging to the group of patients with severe COVID-19.

C-reactive protein is an acute-phase reactant, well known as a marker of systemic inflammation and severe infections [21]. Our results showed that CRP high levels had a significant correlation with CT features for severity in COVID-19 patients. Moreover, in a study conducted by Tan et.al, correlation analysis showed that CRP (R = 0.62; *p* < 0.001) was positively correlated with CT scores [22], as in our study where CRP positively correlates with CT changes (R = 0.40; *p* < 0.001). In similarity with our findings, several studies found that CRP levels correlate with lung lesion at CT scan and disease severity [6,23,24].

IL-6 is a highly pro-inflammatory molecule that can induce other inflammatory cells and mediators that cause pulmonary parenchymal lesions and dyspnea [25]. In the present research, we found that IL-6 is associated with the severity of lesions observed on a chest CT. In addition, Liu et al. showed that IL-6 was positively correlated (R = 0.453; *p* = 0.001) with the bilateral and interstitial pulmonary involvement at CT scan, suggesting that IL-6 can be an ideal disease monitoring marker [26]. Similarly, in our study, IL-6 correlated positively with lung damage observed on a CT scan. In addition, IL-6 showed the highest diagnostic accuracy of lung damage of over 50% among the markers studied, with an AUC of 0.854.

Neutrophil to lymphocyte ratio is an inflammatory biomarker that is associated with poor clinical outcomes in malignant and cardiovascular diseases when elevated [27]. In our study we found that NLR positively correlates with CT features, as well as in a cross-sectional study conducted by Man et.al, NLR correlated positively with severe CT findings along with PLR and eosinophils [28].

Procalcitonin (PCT) is an accurate and specific biomarker for the early diagnosis of bacterial infections [29]. Zhang et.al reported a positive association (*p* < 0.001) between PCT and chest CT scores [30]. In contrast, in our study, procalcitonin has a low positive correlation without statistical significance (*p* = 0.66).

In terms of the likelihood of COVID-19 patients having severe lung damage, all inflammatory markers showed strong predictability. In addition, we recall that patients with previous lung lesions due to lung tumors were excluded, and the odds ratio was adjusted for age and comorbidities. Thus, the adjusted odds of severe lung injury on CT were 9.32 for CRP > 92.5 mg/dL, 5.73 for NLR > 9, 43.24 for IL-6 > 20.75 pg/mL, and 18. 71 for procalcitonin > 0.29 ng/mL, respectively.

Another CT finding was pleural effusions (5.66%). This finding is less common in patients with COVID-19, its incidence being between 2 and 11%. Several studies have found that this finding is commonly seen in critically ill patients with Multisystem Inflammatory Syndrome (MIS) [31,32]. These observations in the literature seem to be in line with the results, with pleural effusions being seen only in patients with severe COVID-19. In addition, another possible cause of pleural effusion is direct invasion of the pleural space by SARS-CoV2 virus, which was found in postmortem studies [33].

One interesting finding that was observed in the results of this study is that there is no statistically significant difference in the length of hospitalization in patients with severe COVID compared to those with non-severe COVID. This non-significant difference can also be based on the difference in mortality, which is higher in severe patients. However, this difference is not significant (*p* = 0.07). In addition, the differences in length of hospitalisation can also be attributed to other factors such as diabetes or the presence of fever [34].

This study had several limitations. First, the study design is retrospective and uses data from a single clinic, the group of patients being relatively small. Second, the CT scans were performed only on admission. Moreover, in this study, negative COVID-19 patients or patients with other infections were not included. Another limitation of the study consisted in the non-use of the most recent and current Berlin criteria for ARDS.

In summary, the inflammatory markers presented in this paper are correlated with lung involvement on chest CT scans and can be used to evaluate the COVID-19 severity. This may be helpful in clinics where CT assessment is not available. Validation of these results by future studies could mean that inflammatory markers can be included in the classification of COVID-19 severity. However, because of the many confounding factors, we believe that CT examination cannot be replaced by assessment of inflammatory markers to determine lung injury in hospitals with CT equipment. Nevertheless, although the association between elevated inflammatory markers and lung lesions may be limited with various confounding factors, it remains as an alternative in the context of no other diagnostic possibility.

## Figures and Tables

**Table 1:** Baseline characteristics of patients (n = 108).

Variables	Overall (n = 106)	Non-Severe COVID-19 (n = 46)	Severe COVID-19 (n = 60)	*p*-Value
Age	69 [61–74]	67 [47.2–72.0]	70.5 [64.5–76.0]	0.01
**Sex**				
Female	58 (54.7%)	22 (47.8%)	36 (60.0%)	0.24
Male	48 (45.3%)	24 (52.2%)	24 (40.0%)	
BMI	28.7 [24.7–33.29]	27.7 [22.5–36.5]	31.1 [25.0–33.2]	0.57
Smoking	38 (35.8%)	14 (30.4%)	24 (40.0%)	0.41
**Comorbidities**				
Hypertension	76 (71.6%)	29 (63.0%)	47 (78.3%)	0.12
Diabetes	56 (52.8%)	23 (50.0%)	33 (55.0%)	0.69
COPD/Asthma	20 (18.9%)	2 (4.35%)	18 (30.0%)	<0.001
Malignancies	18 (17.0%)	7 (15.2%)	11 (18.3%)	0.79
**Laboratory data**				
WBC (×10^9^/L)	7.19 [4.70–9.71]	6.09 [4.35–9.73]	7.26 [4.70–8.65]	0.56
Lymphocytes(×10^9^/L)	0.83 [0.43–1.24]	0.88 [0.42–1.43]	0.81 [0.43–1.12]	0.29
Neutrophils(×10^9^/L)	5.92 [3.59–7.62]	5.51 [3.03–7.50]	5.95 [3.71–8.82]	0.29
Monocytes (×10^9^/L)	0.37 [0.27–0.58]	0.39 [0.29–0.50]	0.36 [0.27–0.67]	0.75
Platelets(×10^9^/L)	190 [146–246]	171 [128–238]	193 [149–271]	0.28
**Inflammatory markers**				
CRP (mg/dL)	69.2 [32.1–125.0]	57.2 [23.2–95.6]	78.0 [43.15–144.0]	0.08
IL-6 (pg/mL)	11.5 [1.93–28.9]	10.4 [1.50–32.7]	11.5 [6.48–25.1]	0.64
NLR	6.60 [4.10–11.7]	5.40 [3.15–10.0]	7.40 [4.40–13.8]	0.08
Procalcitonin (ng/mL)	0.08 [0.05–0.22]	0.08 [0.05–0.18]	0.08 [0.05–0.27]	0.65
**Clinical**				
Temperature (°C)	36.5 [36.1–37.0]	36.4 [36.1–36.7]	36.8 [36.1–37.7]	0.05
Respiratory rate (per minute)	25.5 [21–31]	24.0 [20.0–27.0]	28.5 [23.0–34.0]	<0.001
SpO_2_	93 [87–97]	97 [92–98]	88.5 [82.0–92.0]	<0.001
Hospitalization days	10 [6–15]	9 [5.0–14.0]	11 [6.75–16.0]	0.59
Deaths	28 (26.4%)	8 (17.4%)	20 (33.3%)	0.07
**CT findings**				
Ground-glass opacities	68 (64.2%)	28 (60.9%)	40 (66.7%)	0.54
Pleural effusion	6 (5.66%)	-	6 (10.0%)	-
Consolidation	34 (32.1%)	13 (28.3%)	21 (35.0%)	0.53
Lung Involvement * (Median [IQR])	25 [15–40]	15 [6.25–25.0]	35 [23.7–42.5]	<0.001
Lung Involvement ≥50%	14 (13.2%)	1 (2.2%)	13 (21.7%)	0.003

BMI = Body mass index; COPD = Chronic obstructive pulmonary disease; CRP = C-reactive protein; IL-6 = interleukin-6; NLR = Neutrophil to lymphocyte ratio; SpO_2_ = oxygen saturation measured by pulseoximetry. * Lung involvement was measured in percentages for each patient.

**Table 2:** The optimal cut-offs of CRP, NLR, IL-6, and procalcitonin.

Biomarker	AUC	Optimal Cut-Off	Youden	Sensitivity	Specificity
CRP	0.771	92.5	0.54	85%	68%
NLR	0.727	8	0.54	85%	68%
IL-6	0.854	20.75	0.75	100%	75%
Procalcitonin	0.757	0.29	0.49	64%	85%

CRP = C-reactive protein; IL-6 = interleukin-6; NLR = neutrophil to lymphocyte ratio.

**Table 3:** The adjusted OR in each of the CRP, NLR, IL-6, and procalcitonin.

Biomarker	B	S.E	aOR	*p*-Value	95%CI
CRP > 92.5 (mg/dL)	2.232	0.857	9.322	0.009	1.73–50.01
NLR > 8	1.747	0.839	5.738	0.03	1.10–29.71
IL-6 > 20.75 (pg/mL)	4.177	1.503	43.24	0.004	3.27–571.01
Procalcitonin > 0.29 (ng/mL)	2.929	1.036	18.71	0.005	2.45–142.67

aOR = adjusted odds ratio; CI = confidence interval; CRP = C-reactive protein; IL-6 = interleukin-6; NLR = neutrophil to lymphocyte ratio; S.E = standard error.

## Data Availability

The data sets used and/or analyzed during the present study are available from the corresponding author on reasonable request.
